# Supplementary material for: A Humanized Yeast Phenomic Model of Deoxycytidine Kinase to Predict Genetic Buffering of Nucleoside Analog Cytotoxicity
Source: Genes (Basel). 2019 Sep 30;10(10):770. doi: 10.3390/genes10100770 (PMC6826991; doi:10.3390/genes10100770)
Supplement: Supplementary file 1 [file genes-10-00770-s001.zip › Additional_Files/Additional_File7_GOTermHeatmaps.zip!/Additional_File7_GOTermHeatmaps/mitochondrial_small_ribosomal_subunit.pdf]

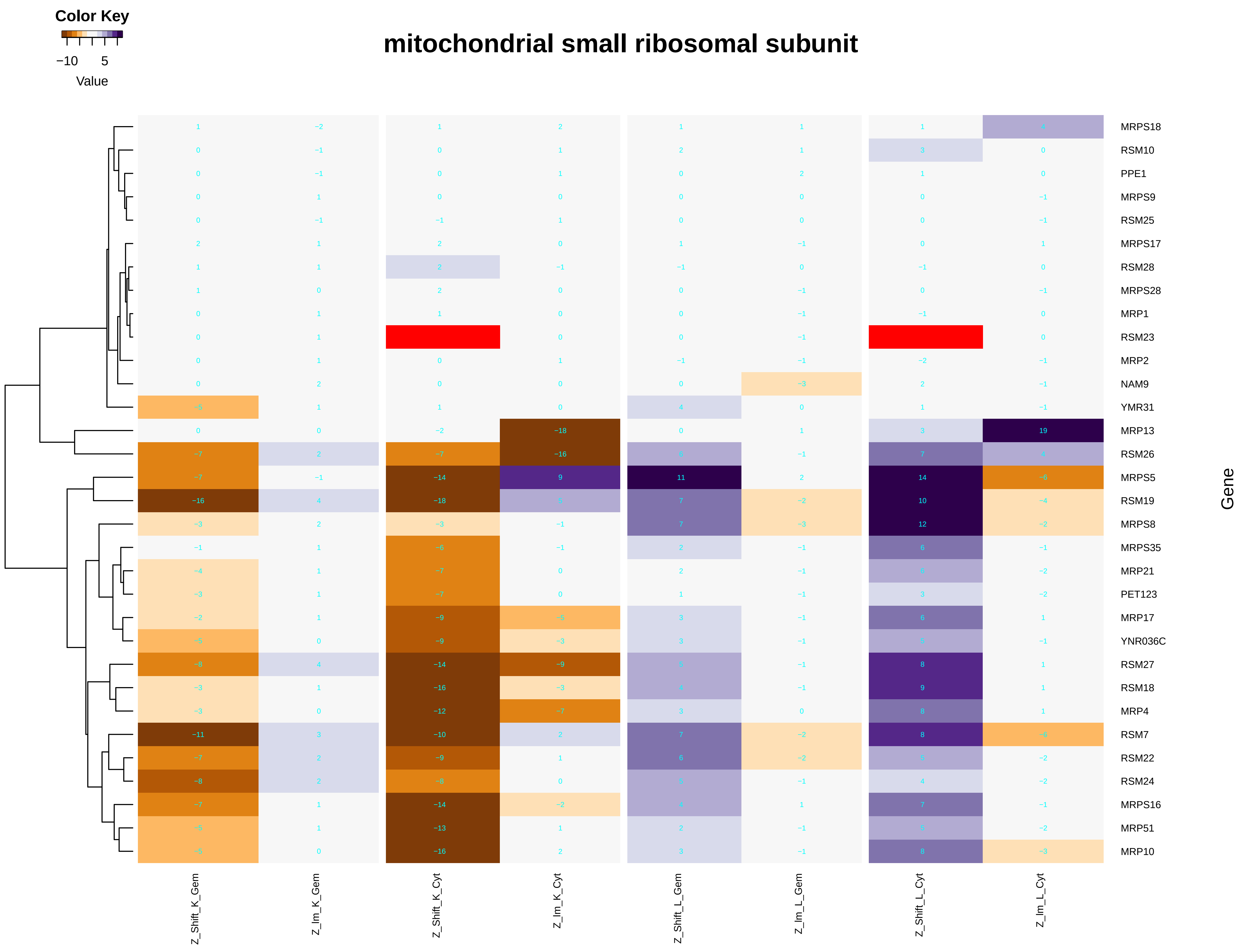

Color Key
−10
5
Value
mitochondrial small ribosomal subunit
MRPS18
RSM10
PPE1
MRPS9
RSM25
MRPS17
RSM28
MRPS28
MRP1
RSM23
MRP2
NAM9
YMR31
MRP13
RSM26
MRPS5
RSM19
MRPS8
MRPS35
MRP21
PET123
MRP17
YNR036C
RSM27
RSM18
MRP4
RSM7
RSM22
RSM24
MRPS16
MRP51
MRP10
Gene
Z_Shift_K_Gem
Z_Im_K_Gem
Z_Shift_K_Cyt
Z_Im_K_Cyt
Z_Shift_L_Gem
Z_Im_L_Gem
Z_Shift_L_Cyt
Z_Im_L_Cyt
